# Supplementary material for: Combination of procalcitonin and C‐reactive protein levels in the early diagnosis of bacterial co‐infections in children with H1N1 influenza
Source: Influenza Other Respir Viruses. 2018 Dec 1;13(2):184–90. doi: 10.1111/irv.12621 (PMC6379630; doi:10.1111/irv.12621)
Supplement: Supplementary file 1 [file IRV-13-184-s001.docx]

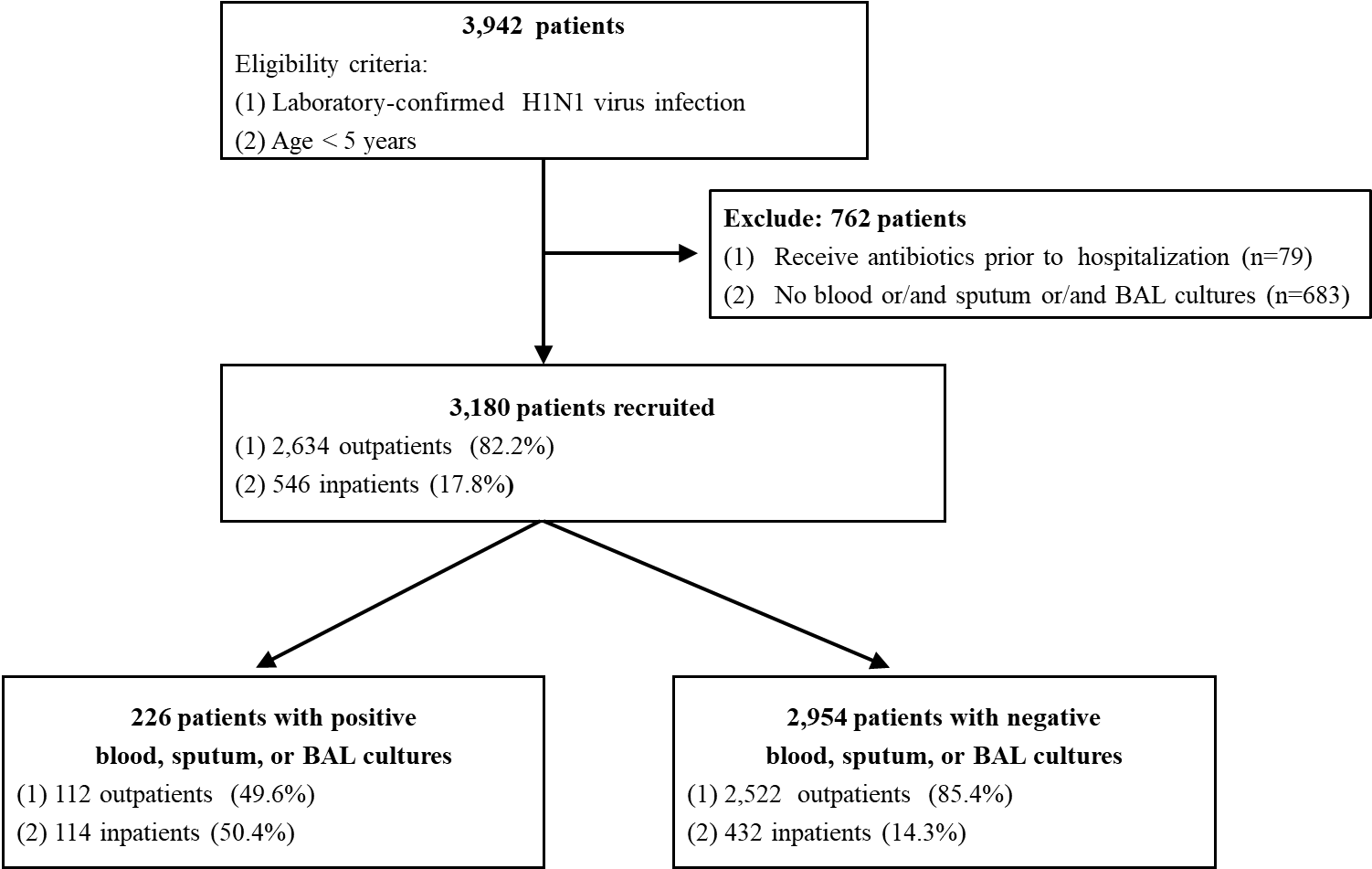


**Figure S1.** Flow chart of patients recruited for the study.

BAL: bronchoalveolar lavage.

**Table S1** Pathogens isolated in patients with H1N1 influenza and a bacterial co-infection

| Pathogens | *n* | % |
| --- | --- | --- |
| *Streptococcus pneumonia* | 82 | 36.3 |
| *Staphylococcus aureus* | 55 | 24.3 |
| *Pseudomonas aeruginosa* | 34 | 15.1 |
| *Haemophilus influenzae* | 21 | 9.3 |
| *Acinetobacter baumannii* | 16 | 7.1 |
| *Moraxella catarrhalis* | 13 | 5.8 |
| *Klebsiella pneumoniae* | 9 | 4.0 |
| *Methicillin-resistant Staphylococcus* | 4 | 1.8 |
